# Supplementary material for: Taxonomic revision of the tarantula genus Aphonopelma Pocock, 1901 (Araneae, Mygalomorphae, Theraphosidae) within the United States
Source: Zookeys. 2016 Feb 4;(560):1–340. doi: 10.3897/zookeys.560.6264 (PMC4768370; doi:10.3897/zookeys.560.6264)
Supplement: Supplementary material 2 — Compressed files (.zip) containing Aphonopelma boxplot and PCA morphospace data. [file zookeys-560-001-s002.zip › Supp2b.zip!/Appendix 2B/Boxplot_morphospace/Aphonopelma_boxplots_males/Pygmy/F4A4_ratio_males.pdf]

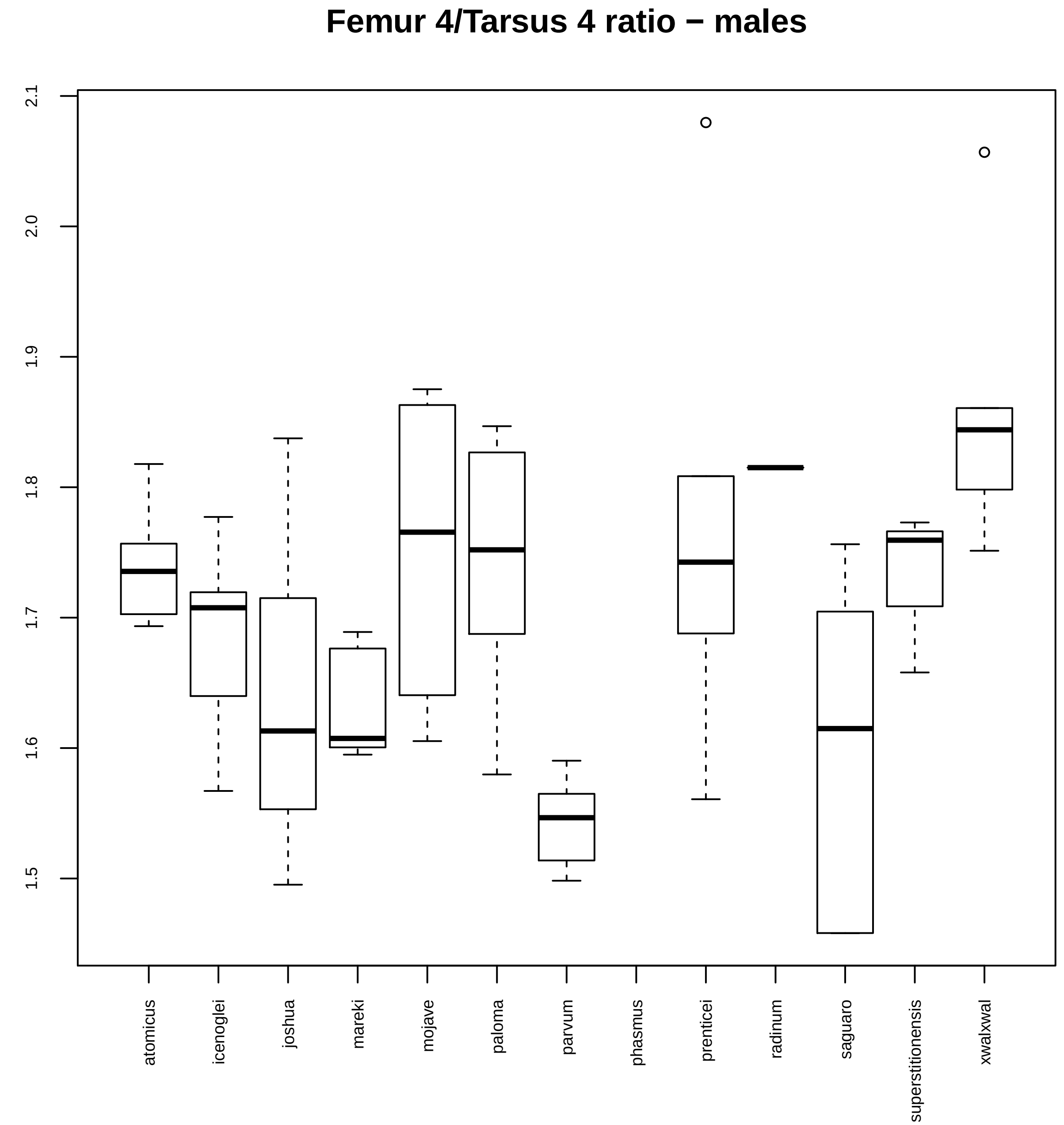

Femur 4/Tarsus 4 ratio − males
1.5
1.6
1.7
1.8
1.9
2.0
2.1
atomicus
icenoglei
joshua
mareki
mojave
paloma
parvum
phasmus
prenticei
radinum
saguaro
superstitionensis
xwalxwal
